# Modulation of the Cellular Expression of Circulating Advanced Glycation End-Product Receptors in Type 2 Diabetic Nephropathy

**DOI:** 10.1155/2010/974681

**Published:** 2011-01-05

**Authors:** Karly C. Sourris, Brooke E. Harcourt, Sally A. Penfold, Felicia Y. T. Yap, Amy L. Morley, Philip E. Morgan, Michael J. Davies, Scott T. Baker, George Jerums, Josephine M. Forbes

**Affiliations:** ^1^Glycation and Diabetes, Diabetes Division, Baker IDI Heart and Diabetes Institute, Melbourne 3004, Australia; ^2^Departments of Immunology and Medicine, Monash University, Alfred Medical Research Education Precinct, Melbourne 3004, Australia; ^3^Heart Research Institute, Newtown, Sydney 2042, Australia; ^4^Faculty of Medicine, University of Sydney, Sydney 2006, Australia; ^5^Endocrine Centre, Austin Health, West Heidelberg, Victoria 3084, Australia; ^6^Department of Medicine, The University of Melbourne, Melbourne 3010, Australia

## Abstract

*Background*. Advanced glycation end-products (AGEs) and their receptors are prominent contributors to diabetic kidney disease. *Methods*. Flow cytometry was used to measure the predictive capacity for kidney impairment of the AGE receptors RAGE, AGE-R1, and AGE-R3 on peripheral blood mononuclear cells (PBMCs) in experimental models of type 2 diabetes (T2DM) fed varied AGE containing diets and in obese type 2 diabetic and control human subjects. *Results*. Diets high in AGE content fed to diabetic mice decreased cell surface RAGE on PBMCs and in type 2 diabetic patients with renal impairment (RI). All diabetic mice had elevated Albumin excretion rates (AERs), and high AGE fed dbdb mice had declining Glomerular filtration rate (GFR). Cell surface AGE-R1 expression was also decreased by high AGE diets and with diabetes in dbdb mice and in humans with RI. PBMC expression of AGE R3 was decreased in diabetic dbdb mice or with a low AGE diet. *Conclusions*. The most predictive PBMC profile for renal disease associated with T2DM was an increase in the cell surface expression of AGE-R1, in the context of a decrease in membranous RAGE expression in humans, which warrants further investigation as a biomarker for progressive DN in larger patient cohorts.

## 1. Introduction

Advanced glycation end products (AGEs) are a heterogeneous and complex group of biochemical modifications, which play an important role in the development of chronic disease processes including the complications of diabetes [1]. AGEs can be produced endogenously as the result of hyperglycaemia or other metabolic imbalances and are also absorbed from foodstuffs, each of which contribute to the body's AGE burden. AGEs may elicit their effects via receptors and binding proteins which are broadly thought to be either inflammatory (e.g. RAGE) or “clearance” receptors (such as AGE-R1, AGE-R3, CD36, Scr-II) [2, 3]. 

The receptor for advanced glycation end products, RAGE, is a transmembrane receptor, expressed on the cell surface of a number of circulating and organ-specific cells including monocytes, macrophages, proximal tubular cells, podocytes, and mesangial cells [4–6]. Gene splicing results in three splice variants, including full-length cell surface RAGE (contains the active binding site, extracellular, and intracellular domains) and endogenous secretory es-RAGE (lacks transmembrane and intracellular domain but contains a secretory tail) [7, 8]. RAGE is also thought to be proteolytically cleaved from the cell surface becoming part of the circulating soluble RAGE pool thus considered to be a decoy receptor [7, 8]. RAGE has been shown to be modulated by both AGEs [9, 10] and by diabetes [4, 11]. 

 AGE-R1 is a type 1 integral membrane protein which is also part of the oligosaccharide transferase complex [12]. AGE-R1 has a short extracellular domain and a long cytoplasmic tail and is thought to be involved in both AGE clearance [13] and apoptosis via the protein p66^shc^ [13]. Cellular expression of AGE-R1 involves both circulating and renal cells including mononuclear, endothelial [14], and mesangial cells [15]. AGE-R1 has been shown to be downregulated by both diets abundantly rich in AGEs [16] and in diabetes [17, 18]. In addition, lymphoblasts from type 1 diabetic patients with progressive diabetic nephropathy have also exhibited a decrease in AGE-R1 [19].

In contrast, AGE-R3 (also known as Galectin-3) is a high affinity AGE-binding protein which is thought to be primarily based in the cytoplasm and nucleus where it binds to AGEs as well regulating cell cycle, replication, and apoptosis [20]. AGE-R3 has been shown to be elevated by AGEs in cultured endothelial cells and within renal tissues in the diabetic milieu [21]. In addition, AGE-R3 has also been shown to be expressed on circulating cells including macrophages, eosinophils, and mast cells [22].

Modulation of AGE-receptor expression has been shown to be important for the development of diabetic nephropathy both clinically and in experimental models [7, 8]. In the present study, we employed flow cytometry as a novel approach to measure AGE-receptor expression in PBMCs in type 2 diabetes using both experimental models fed low or high AGE diets and humans with renal impairment, in order to assess the relevance of circulating cellular AGE receptor expression profiles as biomarkers for diabetic nephropathy.

## 2. Materials and Methods

### 2.1. Experimental Mouse Model of Diabetes

Male *Lepr* ^(+/+)^ *C57BL/KsJ (dbdb*) mice were originally purchased from Jackson Laboratories and randomised at 8–10 weeks of age, to consume diets low in AGE (LA; AIN-93G unbaked) or high in AGE content (HA, AIN-93G baked for 1 hour at 160°C), quantitated by HPLC as previously described [23]. This mouse strain develops diabetic nephropathy in the context of severe metabolic syndrome (hypertension, hyperlipidaemia, obesity, insulin abnormalities) similar to that seen in type 2 diabetes in humans. Male *Lepr* ^(+/−)^ *C57BL/KsJ (dbh) *littermates were followed concurrently (*n* = 10/group) and served as the appropriate control. All groups were followed for 10 weeks. 

At the completion of the study whole blood was isolated by cardiac puncture. All procedures were in accordance with the guidelines set out by the Alfred Medical Research and Education Precinct Animal Ethics Committee and the National Health and Medical Research Council of Australia.

### 2.2. Isolation of Peripheral Blood Mononuclear Cells (PBMCs)

Whole blood (*∼*1 mL), was collected in heparinised tubes (20 IU Sodium Heparin). Blood was centrifuged at 500 × g, for 5 mins, the plasma removed, and its volume replaced with 0.9% (w/v) saline. The whole blood suspension was then diluted to a final volume of 6 mL, underlaid with 3 mLs of Ficoll-Paque (Amersham, Upsalla Sweden), and centrifuged at 800 × g for 25 mins to separate the peripheral blood mononuclear cells. PBMCs were carefully removed from the interface and transferred to a clean tube and washed twice in excess phosphate buffered saline (PBS, pH 7.4 without Ca^++^). Finally, PBMCs from three mice from the same group were pooled and diluted to a final concentration of 1 × 10^7^ cells/ml in PBSF (PBS, 2.5% (v/v) FBS, 0.1% (v/v) NaN_3_).

To isolate human PBMCs, baseline blood samples were collected from participants who had provided written informed consent prior to participation in the Diabesity study conducted by the Endocrinology Department at Austin Health. Twenty-four-hour albumin excretion rates were determined by radioimmunoassay, isotopic GFR using ^99^Tc-DTPA, and HbA_1C_ by HPLC as previously described in [24]. The protocol was approved by the Human Ethics Committee of Austin Health and complied with the Declaration of Helsinki, 2004. Whole blood (8 mLs) from control (obese, nondiabetic; *n* = 5), diabetic (obese, diabetic, *n* = 15) and diabetic subjects with renal impairment (RI) (*n* = 7; Table 2) was collected in BD CPT (362761, BD Australia) tubes and processed as per manufacturer's instructions.

### 2.3. Flow Cytometric Staining for Advanced Glycation End-Product Receptors on PBMCs

PBMCs (1 × 10^6^ cells/tube), isolated from experimental models and diabetic patients as described above, were stained for cell surface receptors with 10 *μ*L goat-anti-RAGE (N-16; Santa Cruz, USA), AGE-R1 10 *μ*L (mouse) or 5 *μ*L (human) OST-48 H-300, Santa Cruz, USA) for 30 mins at room temperature. Cells were then washed with excess PBSF and centrifuged (500 g, 5 min, RT). Supernatant was removed, and the cell pellets were resuspended in 100 *μ*L PBSF. antigoat (F (ab'2) Chemicon, USA; 1 : 250) and Antirabbit (AP322F, Chemicon, USA; 1 : 500) FITC- conjugated secondary antibodies were added to the appropriate tubes and incubated at room temperature, for a further 30 mins in the dark. After washing, the supernatant was removed and cell pellet resuspended in FACS fixative (2% w/v paraformaldehyde in PBSF). Cells were fixed for 20 mins 4°C and washed with excess PBSF. Other tubes of PBMCs were also concurrently stained with an isotype control FITC-conjugated Rat IgG_2bk_ (556923, BD Pharmingen, USA, mouse PBMC) or FITC-conjugated Mouse IgG_1 _(349526, BD Pharmingen, USA) (human PBMC) and CD45 FITC (FITC-conjugated rat antimouse CD45, 553079; FITC-conjugated Mouse antihuman CD45, 555482) for the determination of background binding and cell type, respectively. 

For intracellular markers, following fixation PBMCs were washed and cell pellet resuspended in 100 *μ*L 0.3% w/v Saponin (diluted in PBSF, S4521, Sigma-Aldrich) to permeabilise the cells. Cells were stained for intracellular expression of AGE-R1 (OST48 (H-300) Santa Cruz, USA) and AGE-R3 (5 *μ*L for mouse and 10 *μ*L for human, AF1197, R&D Systems, USA), 30 mins 4°C. Cells were washed once again and resuspended in saponin and stained with their appropriate secondary antibody, antigoat (F (ab'2) Chemicon, USA; 1 : 250), and antirabbit (AP322F, Chemicon, USA; 1 : 500), at 4°C for 30 min, in the dark. At completion of the incubation, cells were washed once again with excess PBSF and cell pellet resuspended in FACs fixative. For both intra- and extracellular receptors, a minimum of 10,000 events were acquired on an FACsCalibur, BD Biosciences, USA. Cells were gated according to their forward and side-scatter properties. Isotype and CD45 FITC controls were also employed for compensation of the instrument. Data were analysed using WIN MDI v 2.9 (http://ptf.com/winmdi/winmdi+2.9+download/). Positive cells were identified using histogram analysis and fluorescence relative to the secondary antibody alone control.

### 2.4. Measurement of N^*ε*^-Carboxymethyllysine (CML) in Plasma, Urine, and Rodent Chow

CML was measured in mouse plasma (1 : 8000) and urine (1 : 2) and in human plasma (1 : 25600) at their respective dilutions, using an in-house ELISA that has been previously described in [18]. In addition, *N^*ε*^*-carboxymethyllysine (CML) levels were measured in low and high AGE diets using HPLC and standardised to the amino acid serine (Ser) in that same sample as previously described in [23].

### 2.5. Measurement of Thiamine in Rodent Diet

Thiamine content was quantitated in 100 gms of low and high AGE diets by Pathwest Laboratories, Nedlands, WA, Australia. 

### 2.6. Statistical Analysis

Data are expressed as means ± SD, unless otherwise stated. Analyses of data were performed by ANOVA followed by post hoc analysis using Tukey's test or nonparametric *t*-tests. Data for albuminuria were not normally distributed and therefore analysed after logarithmic transformation. *P* < .05 was considered statistically significant.

## 3. Results

### 3.1. Experimental Model of Metabolic Syndrome and Type 2 Diabetes (the dbdb Mouse)

Male *Lepr* ^(+/+)^ *C57BL/KsJ (dbdb*) mice were used as a model representative of type 2 diabetes. Analysis of the low and high AGE diets administered to these mice revealed that the CML content was 90 or 370 nmoles CML per mole of serine, respectively. In addition, Thiamine content was found to be 2.61 and 3.51 *μ*g/g in the low and high AGE diets. Renal and metabolic parameters from this mouse model are shown in Table 1. Dbdb mice had elevated blood glucose, renal hypertrophy and increased urinary albumin excretion when compared with dbh mice irrespective of diet. In addition, a decline in creatinine clearance and increased plasma cholesterol were seen in high AGE fed dbdb mice when compared with dbdb mice fed a low AGE diet (Table 1). Serum CML concentrations were not altered among any groups. In contrast however, urinary CML concentrations were significantly elevated in diabetic dbdb mice (dbh LA, 0.0025 ± 0.0021 versus dbdb LA, 5.654 ± 5.116 *μ*mol/mol/lysine/24 hrs; *P* = .0476; dbh HA, 0.0155 ± 0.0120 versus dbdb HA, 3.517 ± 3.588 *μ*mol/mol lysine/24 hr, *P* = .0364).

**Figure 1:**
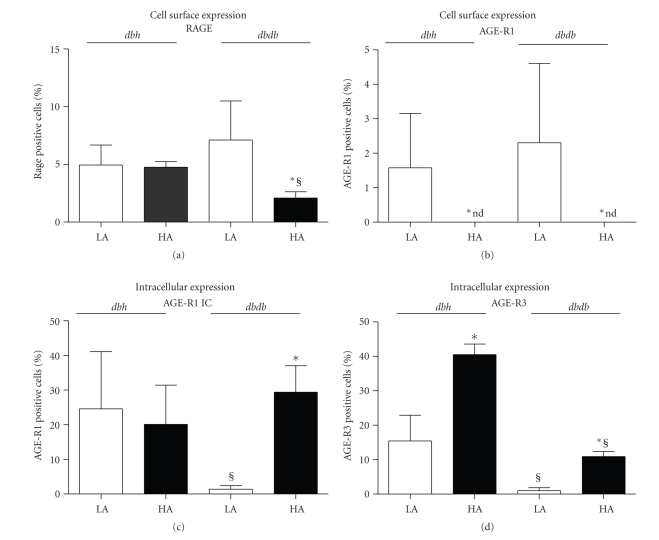
Flow cytometric analysis for the cell surface expression of (a) RAGE, (b) AGE-R1 and intracellular levels of (c) AGE-R1 and (d) AGE-R3 on PBMCs in *dbh and dbdb mice at week 20 of age.* Empty bars: low AGE diet (LA) and filled bars: high AGE (HA) groups. *P < .05 versus corresponding low AGE group, ^§^ *P* < .05 versus dbh group within the same diet.

In heterozygous dbh mice, a diet high in AGE did not alter cell-surface expression of RAGE on PBMCs (Figure 1(a)). By contrast, there was a significant loss of cell surface RAGE expression on PBMCs from high AGE fed dbdb mice as compared with low AGE fed dbdb mice (Figure 1(a)). High AGE diets significantly declined the PBMC cell surface expression of AGE-R1 in both dbdb and dbh mice (Figure 1(b)), which was not altered by diabetes. Intracellular levels of AGE-R1 were not altered in the dbh mice by a high AGE diet (Figure 1(c)). However, dbdb mice fed a low AGE diet had significantly lower intracellular expression of AGER1 in PBMCs as compared to both high AGE fed dbdb mice and low AGE fed dbh mice (Figure 1(c)). High AGE dietary intake increased the expression of AGE-R3 in dbh and to a lesser extent in dbdb mice. Overall, diabetic dbdb mice exhibited significantly lower levels of AGE-R3 relative to dbh counterparts.

### 3.2. AGE-Receptors in PBMCs from Type 2 Diabetic Subjects

 We next investigated AGE-receptor expression on PBMCs from control, diabetic, and diabetic subjects with renal impairment, all of whom were obese. Renal and metabolic parameters for these subjects are shown in Table 2. Type 2 diabetic individuals had a significant increase in HbA_1c_ and albuminuria tended to increase in concert with renal impairment although this did not reach statistical significance (*P* = .07). Diabetic patients with either a decline in isotopic GFR to a level <60 mL/min/1.73 m^2^ or an albumin excretion rate >200 *μ*g/min were included as having early renal impairment (Table 2). Diabetic individuals with renal impairment also had lower diastolic blood pressure.

**Figure 2:**
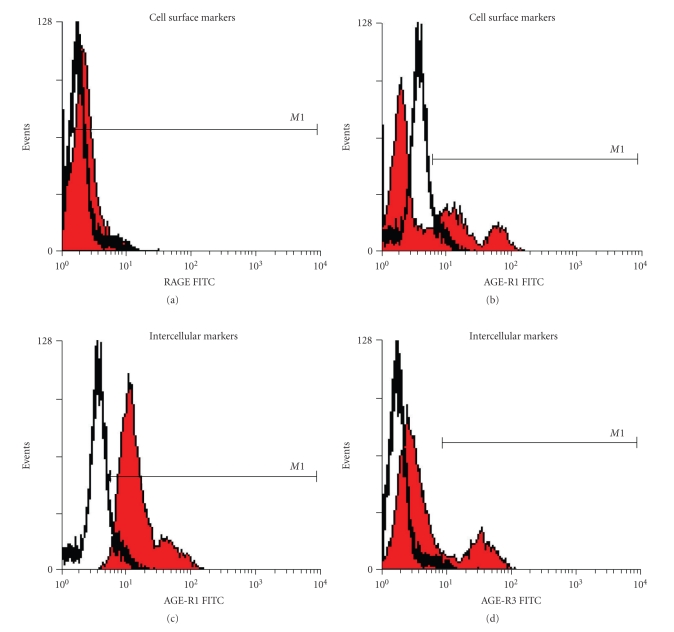
Representative histograms of flow cytometric analysis of AGE receptors in PBMCs isolated from type 2 diabetic patients. Cell surface expression of (a) RAGE and (b) AGE-R1. Intracellular (c) AGE-R1 and (d) AGE-R3 within PBMCs. Receptor positive cells (*M*1 filled histogram) were identified as those which fluoresced above their relative secondary antibody (empty histogram).

**Figure 3:**
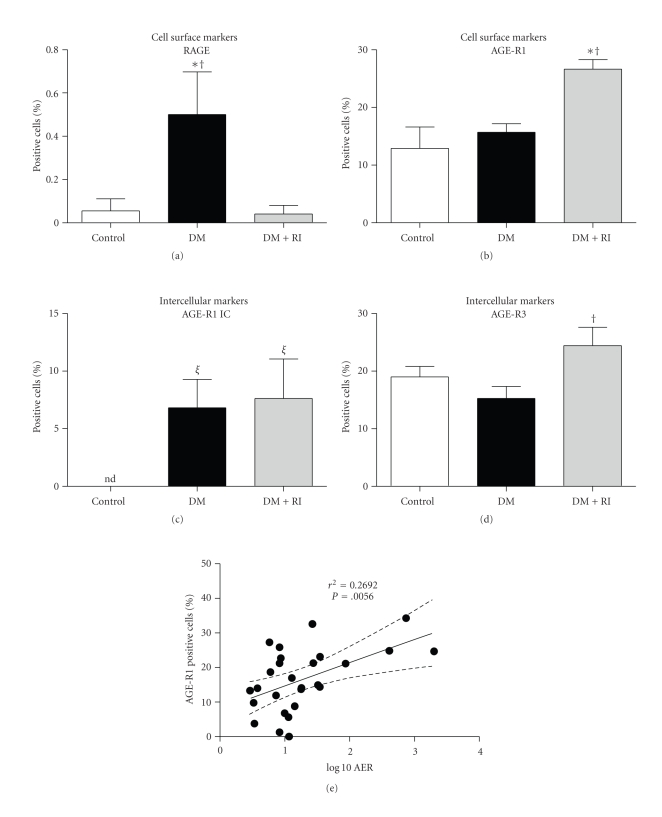
Flow cytometric analysis for the cell surface expression of (a) RAGE and (b) AGER-1 and intracellular levels of (c) AGE-R1 and (d) AGE-R3 on PBMCs of human control, diabetic (DM) and diabetic individuals with renal impairment (DM + RI). (e) Positive correlation between cell surface expression of AGE-R1 and Albumin Excretion Rate (AER), *P* = .0056. Empty bars: control, grey bars: diabetes, and black bars are diabetes with renal impairment (iGFR < 90) groups. nd: not detected. *P < .05 versus control, ^†^ *P* < .05 versus DM, ^*ξ*^ *P* < .0001 versus control (*n* = 5–10/group).

Consistent with the experimental models, cell surface expression of RAGE and AGE-R1, in addition to intracellular levels of AGE-R1 and AGE-R3, was readily measured by flow cytometry in human PBMCs (Figure 2). Diabetes induced a significant increase in cell surface RAGE expression on PBMCs, which was significantly reduced in diabetic patients with renal impairment (Figure 3(a)). By contrast, extracellular AGE-R1 expression was not affected by diabetes per se; however, PBMCs from diabetic subjects with diabetes and renal impairment had a significant increase in this receptor (Figure 3(b)). Diabetes increased the intracellular PBMC expression of AGE-R1 (Figure 3(c)), while AGE-R3 expression was elevated in type 2 diabetic patients with renal impairment, consistent with cell surface AGE-R1 expression.

## 4. Discussion

In the present study we have identified that the most predictive PBMC profile for progressive renal disease in type 2 diabetes in humans was an increase in the cell surface expression of AGE-R1 in the context of a decrease in cell surface RAGE. However, in contrast to a number of previous studies [10, 25], we have not identified increases in circulating AGE modified protein concentrations in association with early renal disease, in the diabetic mouse model used, nor in type 2 diabetic individuals. In addition, there appears to be no association among the expression of AGE receptors studied on PBMCs and circulating CML concentrations in this study.

### 4.1. Modulation of Circulating Levels of AGEs

Whilst CML is one of the most well-characterised AGEs to date, there are numerous others which have also been well characterised including N-carboxyethyllysine (CEL), pentosidine, imidazole, glyoxal-lysine dimer, pyrraline, and methylglyoxal (MGO). Given that HPLC analysis of the rodent chow failed to detect measurable levels of other AGEs apart from CML, we chose to focus on that modification. In addition, the lack of differences in thiamine levels between baked and unbaked chow suggests that the dietary effects demonstrated in the present study are primarily via differences in CML levels rather than via depletion of vitamin B. The ELISA used in the present study for CML only recognises protein-bound CML (no CML modified peptides or free CML as are often recognised in many of the assays used previously), which likely explains the discrepancies between the present study and others. The plasma to urinary CML ratio is also important when considering the overall body AGE burden in humans and in diabetic rodent models. Indeed, type 2 diabetic dbdb mice have vastly elevated urinary excretion of CML modified proteins in the absence of increases in serum concentrations of CML. Therefore the kidneys of diabetic mice in the present study are likely to be exposed to more CML modified proteins (AGEs) than those kidneys from nondiabetic mice. Since there were also no differences in serum concentrations from obese type 2 diabetic subjects with early renal impairment, it is prudent to suggest that these individuals may also have an elevated urinary excretion of CML, although this was not able to be measured in the present study. Also, rodent models are often homogeneous within groups, especially for markers of renal function such as creatinine clearance due to their genetic background. However, there was significant heterogeneity across the type 2 diabetic patients with renal impairment with respect to iGFR, with some patients showing iGFR > 130, often characteristic of obesity and about half having an iGFR < 60 ml/min/1.73 m^2^_,_ indicative of more significant renal impairment. The greatest differences seen within this group were in the spread of albuminuria which may have been due to the early nature of the renal impairment or due to the fact that in some studies in humans, urinary albumin excretion does not correlate with progressive renal disease [26, 27]. The concentration of CML-modified proteins in type 2 diabetes is likely to be altered by a number of parameters independent of glucose such as insulin levels, food intake [28], and hyperlipidaemia [29], and therefore CML modified proteins may not be directly responsible for all of the changes observed in the AGE receptors in type 2 diabetes.

### 4.2. Modulation of AGE Receptors

Importantly, in the present study, changes seen in cellular expression of AGE receptors were closely associated with renal impairment in type 2 diabetes. There has been previous studies using dietary AGEs which demonstrate modulation of cellular RAGE expression. Administration of diets low in AGE content has been associated with reductions in RAGE expression which are paralleled by improvements in renal function and insulin sensitivity in mice [30, 31]. In contrast, in the present study we found that administration of a diet low in AGE to type 2 diabetic mice was associated with increases in RAGE expression on PBMCs along with significant improvements in renal function. The discrepancy seen with these findings may be the result of the methodologies employed. In our present study we have measured cell-surface RAGE expression on circulating cells by flow cytometry, whilst other studies have employed immunoblotting, which would measure all isoforms of RAGE including soluble RAGE, in addition to both cell surface and cytoplasmic RAGE cellular content. Indeed our findings suggest that the use of RAGE antagonists, which are currently under development for diabetic complications (Phase 2B clinical trials), may warrant further consideration for those patients with early renal impairment, given that there may be some temporal differences in RAGE expression over the course of the development and progression of diabetic renal disease.

A decline in AGE-R1 expression on PBMCs has been reported in various chronic diseases [19, 30]. In addition, dietary AGEs have been shown to modulate renal AGE-R1 expression in mice, although these studies have also measured AGE-R1 expression by western immunoblot analysis [9, 30], which does not differentiate between cell surface or intracellular expression of these receptors. In particular, this is important for AGE-R1 which has specific intracellular function as part of the oligosaccharyltransferase complex involved in N-glycosylation [32]. In our present study, high-AGE diets induced a decrease in cell surface AGE-R1 in dbdb and dbh mice, our model of type 2 diabetes, independent to glucose. In addition, the type 2 diabetic human subjects studied with renal impairment clearly showed an increase in AGE-R1 expression on PBMCs, and this correlated with albumin excretion rate. Indeed, we have demonstrated, for the first time, that the cell-surface and intracellular expression patterns of AGE-R1 in circulating PBMCs differ and in fact may be opposite to what has been previously reported regarding diabetes-induced early renal impairment in humans. This may be due to the fact that the patient cohort studied had very early renal disease as compared to the type 2 diabetic mouse model which was used in the present study. 

We have also demonstrated an increase in AGE-R3 expression in PBMCs in diabetic humans with renal impairment, consistent with previous reports [33, 34]. Indeed, previous studies in diabetic AGE-R3 knockout mice have shown severe renal dysfunction [35]. The interrelationship among AGE clearance receptors in diabetic nephropathy remains to be fully elucidated, although it is interesting to note that, in the present study, AGE-R1 and AGE-R3 were each increased with renal impairment in diabetes in humans, in addition to high AGE feeding in our mouse models. This is likely representative of a higher AGE load which requires clearance and detoxification as a protective mechanism. Importantly, the decrease in AGE-R3 seen in the high AGE fed dbdb group may be the result of the advanced renal injury. By contrast the failure to demonstrate this decrease in the patients with renal impairment could be due to the absence of this advanced renal injury in this group suggesting that there may be an initial increase in AGE-R3 expression as a protective mechanism to clear the increased AGE-load.

In conclusion, our findings have clearly demonstrated differences between cell-surface and intracellular patterns of expression of circulating AGE receptors and renal function in diabetes. The most predictive PBMC profile for renal disease in diabetes in humans was an increase in the cell surface expression of the AGE-R1, in the context of a decrease in RAGE, which warrants further investigation as a biomarker for progressive DN in large patient cohorts since these markers were also associated with creatinine clearance in type 2 diabetes. Further investigation is warranted to determine whether this noninvasive approach may be employed to rationalise biomarkers for progressive DN in large patient cohorts in type 2 diabetic patients.

## Figures and Tables

**Table 1:** Renal and metabolic parameters in an experimental model of metabolic syndrome and type 2 diabetes the dbdb mouse followed from weeks 10 to 20 of age. Dbh: non diabetic control mice, dbdb: diabetic mice.

	*N*	Plasma glucose (mmol/L)	Total cholesterol (mmol/L)	Circulating AGE (*μ*mol/mol lysine)	Creatinine clearance (ml/min/m^2^)	Albumin excretion rate (*μ*g/24 hr)	Kidney to body weight ratio (% ×10^−3^))
dbh Low AGE	10	19.6 ± 4.9	1.5 ± 0.2	304.1 ± 106.8	17.2 ± 1.4	226.5 ± 34.6	1.13 ± 0.16
dbh High AGE	10	18.8 ± 5.3	1.4 ± 0.2	357.3 ± 88.2	24.2 ± 6.3^§^	151.0 ± 12.9	1.22 ± 0.13
dbdb Low AGE	10	38.0 ± 8.5^$^	1.5 ± 0.5	353.3 ± 136.1	20.1 ± 15.4	2608.0 ± 791.1^$^	1.62 ± 0.27^$^
dbdb High AGE	10	35.7 ± 6.3^$^	1.8 ± 0.5^$^	284.8 ± 69.1	13.4 ± 8.0^#$^	2334.3 ± 520.2^$^	1.63 ± 0.24^$^

^§^ *P* < .05 versus dbh Low AGE, ^#^ *P* < .05 versus dbdb Low AGE, ^$^ *P* < .05 versus corresponding dbh group.

**Table 2:** Metabolic and renal parameters in control and type 2 diabetic subjects. BMI: body mass index, SBP: systolic blood pressure, and DBP: diastolic blood pressure.

	Control	Diabetes	Diabetes + RI
*N*	5	15	7
HbA_1c_ (%)	5.6 ± 0.4	8.1 ± 1.2*	7.4 ± 0.8*
BMI (kg/m^2^)	34.5 ± 4.9	39.9 ± 6.8	39.9 ± 6.6
AER (*μ*g/min)	11.7 ± 5.9 (2.9–32.9)	21.9 ± 21.7 (3.8–86.6)	356.7 ± 667.7 (3.3–1998.0)
iGFR (mL/min/1.73 sqm)	131.0 ± 28.4	114.2 ± 16.8	93.0 ± 38.6^†^
SBP (mmHg)	135.0 ± 7.9	143.3 ± 14.8	132.1 ± 16.6
DBP (mmHg)	86.0 ± 8.9	86.7 ± 9.8	73.6 ± 14.4^†^
Circulating CML (*μ*mol/mol lysine)	955.6 ± 129.7	916.2 ± 207.7	885.8 ± 119.3

*P < .05 versus control, ^†^ *P* < .05 versus diabetes, ^§^ *P* = .07 (ns) versus diabetes.
